# *Brucella suis* Δ*mapB* outer membrane vesicles as an acellular vaccine against systemic and mucosal *B. suis* infection

**DOI:** 10.3389/fimmu.2024.1501791

**Published:** 2025-01-20

**Authors:** Florencia Muñoz González, Magali G. Bialer, Maria L. Cerutti, Silvia M. Estein, Lila Y. Ramis, Pablo C. Baldi, Ángeles Zorreguieta, Mariana C. Ferrero

**Affiliations:** ^1^ Facultad de Farmacia y Bioquímica, Cátedra de Inmunología, Universidad de Buenos Aires, Buenos Aires, Argentina; ^2^ Instituto de Estudios de la Inmunidad Humoral (IDEHU), CONICET-Universidad de Buenos Aires, Buenos Aires, Argentina; ^3^ Fundación Instituto Leloir (FIL), IIBBA-CONICET (CONICET-FIL), Buenos Aires, Argentina; ^4^ Centro de Rediseño e Ingeniería de proteínas (CRIP), Universidad Nacional de San Martín, Buenos Aires, Argentina; ^5^ Laboratorio de Inmunología, Departamento de Sanidad Animal y Medicina Preventiva (SAMP), Centro de Investigación Veterinaria Tandil (CIVETAN-CONICET-CICPBA), Facultad de Ciencias Veterinarias (FCV), Universidad Nacional del Centro de la Provincia de Buenos Aires (UNCPBA), Tandil, Buenos Aires, Argentina; ^6^ Departamento de Química Biológica, Facultad de Ciencias Exactas y Naturales, Universidad de Buenos Aires, Buenos Aires, Argentina

**Keywords:** outer membrane vesicles, *Brucella suis*, vaccine, TAM, respiratory infection

## Abstract

**Introduction:**

Swine brucellosis, caused by *Brucella suis*, is a worldwide infectious zoonotic disease. Currently, there are no available human or porcine vaccines to protect against *B. suis* infection, which is primarily acquired through the mucosa. We recently described *B. suis* MapB, the homologous protein of TamB, the inner membrane component of the TAM system. Our findings indicate that MapB is involved in bacterial cell envelope homeostasis. In this study, we characterize the outer membrane vesicles (OMVs) of *B. suis* 1330 (wt) and those of *B. suis* Δ*mapB* (Δ*mapB*) mutant strain and evaluate their vaccine potential in mice.

**Methods:**

OMVs were isolated using the ultracentrifugation method and characterized through electron microscopy, Dynamic Light Scattering, SDS-PAGE and proteomics. Immunogenicity was assessed by intramuscular immunization of mice with wt OMVs or Δ*mapB* OMVs, followed by the measurement of antigen-specific antibody levels and functional assays to evaluate the protective capacity of the antibodies. Cellular immunity was assessed by characterizing cytokine secretion through ELISA after *in vitro* stimulation of spleen cells with heat-killed *B. suis*. To determine the level of protection conferred by immunization, mice were challenged with virulent *B. suis* via intraperitoneal or intratracheal routes, and the bacterial load was quantified post-challenge.

**Results:**

Dynamic Light Scattering of the OMVs from both strains revealed the presence of spherical structures of 90-130 nm. Proteomic analysis identified 94 and 95 proteins in the wt and Δ*mapB* OMVs, respectively, including several known *Brucella* immunogens. Both OMVs showed immunoreactivity with sera from *Brucella*-infected pigs. Intramuscular immunization of mice with both OMVs induced antigen-specific IgG in serum, with the Δ*mapB* OMVs group showing higher titers compared to the wt OMVs group. Serum antibodies from both OMVs groups reduced *B. suis* adherence and invasion of lung epithelial cells and enhanced its phagocytosis by macrophages. Upon *in vitro* antigen stimulation, spleen cells from mice immunized with Δ*mapB* OMVs secreted higher levels of interleukin-17 and especially gamma interferon compared to cells from mice immunized with wt OMVs, suggesting the induction of a stronger T helper 1 response in the Δ*mapB* OMVs group. While immunization with both wt and Δ*mapB* OMVs achieved the same level of protection following intratracheal infection with *B. suis* (p<0.01), immunization with Δ*mapB* OMVs provided higher levels of protection against intraperitoneal infection.

**Discussion:**

Overall, these results demonstrate that the *B. suis* Δ*mapB* OMVs are immunogenic and capable of inducing both cellular and humoral immune responses that protect against mucosal and systemic *B. suis* challenges.

## Introduction

Brucellosis, caused by Gram-negative bacteria of the genus *Brucella*, is a worldwide zoonotic disease that leads to abortions and infertility in various domestic animals and causes a chronic, debilitating illness in humans (1). To date, twelve *Brucella* species with different host specificities have been identified (2–5), among which *B. abortus* (mainly infecting cattle), *B. melitensis* (goats and sheep), and *B. suis* (swine) are the species with the highest zoonotic potential.

*B. suis* is prevalent among domesticated pigs in regions such as Asia and Latin America. Although this pathogen has been nearly eliminated from commercial herds in certain areas, it persists among wild or feral swine in various regions, including North America and Europe. This persistence poses challenges for controlling brucellosis, particularly for domesticated pigs kept in outdoor environments. Moreover, clinical cases occasionally occur in hunting dogs and individuals who hunt wild boars. In some instances wild suids also transmit the infection to other livestock, such as cattle, thereby increasing the risk to human health (6).

Human infection can be acquired by direct contact with *Brucella*-infected animals or their products, consumption of unpasteurized dairy products, or inhalation of contaminated aerosols (1, 7, 8)​. *B. suis* poses a major risk especially for slaughterhouse workers, who may become infected through aerosolized bacteria during slaughter (9). There is currently no approved vaccine for human use, so prevention and/or eradication of the disease in domestic animals are the main strategies to prevent human brucellosis.

Available brucellosis vaccines approved for domestic ruminants are based on live attenuated strains of *B. abortus* (*B. abortus* S19, *B. abortus* RB51) and *B. melitensis* (*B. melitensis* Rev.1), which can still cause disease in humans (10–13). Currently, no licensed and effective vaccines are available to prevent *B. suis* infection in swine in most countries where the disease is endemic. Due to the zoonotic potential of *B. suis* and the high prevalence of the infection among domestic and feral pigs in certain areas, it is crucial to develop novel and safe vaccines for swine brucellosis.

Gram-negative bacteria release, at all stages of growth, nanovesicles called Outer Membrane Vesicles (OMVs), which range in size from 20 to 300 nm (14, 15). These vesicles are produced by the blebbing of the Outer Membrane (OM), with the closure of the evaginated membrane at the time of release. They are composed mainly of OM molecules along with periplasmic and cytoplasmic components and nucleic acids (15–17). In recent years, several studies have evaluated OMVs from various Gram-negative bacteria as acellular vaccines (17–24). These vesicles possess attractive characteristics as vaccines, including their content of diverse bacterial antigens in their natural conformation, their non-infectious nature, and the presence of numerous pathogen-associated molecular patterns (PAMPs) on their surface, which confer them immunostimulatory capacity and, in some cases, self-adjuvant activity (15–17).

Several zoonotic strains of *Brucella* spontaneously release OMVs from their surface into the environment during growth. Chemical and proteomic studies revealed that these OMVs contain several molecules related to immune protection against *Brucella* infection, including Outer Membrane Protein 16 (Omp16), Omp19, SodC, Omp25, Omp31, among others (25–32). Parenteral vaccination of mice with OMVs from different *B. melitensis* strains has been shown to reduce the *B. melitensis* load after challenge (25). Furthermore, vaccination with OMVs from *B. abortus* 2308 and *B. abortus* RB51 protected mice from parenteral infection with virulent *B. abortus* 2308 (26). It remains unknown whether OMVs from *B. suis* may constitute an effective acellular vaccine against *B. suis* infection, and whether immunization with these vesicles protects against mucosally acquired infection.

It has been described in Gammaproteobacteria that the TamA and TamB proteins together form the Translocation and Assembly Module (TAM) (33). Initially, this system was associated with the translocation of autotransporters and other β-barrel proteins across the OM (33). More recently it has also been linked to phospholipid homeostasis (34, 35). The *B. suis* MapB protein is the homolog of TamB. We previously demonstrated that the *B. suis* Δ*mapB* mutant exhibited envelope-associated phenotypes. This mutant strain showed increased sensitivity to 0.1% Triton X-100 and the cationic lipopeptide polymyxin B, and it showed an attenuated phenotype in cultured macrophages and the mouse model of infection. In addition, the Δ*mapB* strain displayed defects in cell division, with many cells showing aberrant shapes, such as multiple septa or Y-shaped morphologies. Proteomic analyses of total membrane proteins revealed that the Δ*mapB* mutant exhibits a reduction in the relative amounts of a subset of proteins, including members of the Omp25/31 family, compared to the wild-type strain (36).

It has been suggested that as bacteria grow and recycle their outer membranes, their cell walls tend to release more OMVs, particularly at the dividing septa. Moreover, it is well documented that mutants of Omps linked to the peptidoglycan layer, such as OmpA, Lpp, TolB, and Pal, exhibit an enhanced blebbing process (37–40). Therefore, we hypothesized that the presence of multiple ectopic septa and the altered levels of peptidoglycan-interacting Omps in the Δ*mapB* strain of *B. suis* could result in an increased production of OMVs and/or changes in the antigen composition of these vesicles, potentially enhancing their efficacy as vaccine antigens.

In this study, we characterized the wt and Δ*mapB* OMVs and evaluated their immunogenicity and protective capacity against systemic and mucosal *B. suis* challenges.

## Material and methods

### OMVs isolation

OMVs were obtained as previously described, with minor modifications (41). Briefly, *B. suis* 1330 and *B. suis* Δ*mapB* were grown in Gerhardt Wilson minimal medium supplemented with 1% yeast extract for 48 h at 37°C under agitation and harvested by centrifugation during the early stationary phase of growth. The cell-free supernatant was filtered through 0.22 µm-pore-size filters to remove any remaining bacteria. Plating on Tryptic Soy Agar (TSA) confirmed the absence of viable *B. suis* in the filtered supernatant. The filtered supernatants were ultracentrifuged for 5 h at 100,000 x g and 4°C. The pellet containing the OMVs was resuspended in 1 ml of sterile PBS, and the protein content was quantified using the bicinchoninic acid (BCA) method (Pierce) according to the manufacturer’s instructions. OMVs were aliquoted and stored at -20°C until use. All manipulations of viable *Brucella* were performed in a biosafety level 3 (BSL3) laboratory at the Leloir Institute Foundation-IIBBA, CONICET.

### SDS-PAGE and Western Blot analysis

Isolated OMVs were separated by SDS-PAGE 15% polyacrylamide gels, stained with AgNO_3_, or transferred to a nitrocellulose membrane. Immunochemistry analysis for *Brucella* smooth lipopolysaccharide (S-LPS), Omp31, Omp25, Omp16, Omp19, and Omp10 was performed as described (36)​.

### Transmission electron microscopy (TEM)

OMVs aliquots were fixed with glutaraldehyde 2.5% and negatively strained with 2% uranyl acetate, 2% phosphotungstic acid and analyzed in a Zeiss 10 transmission electron microscope (LANAIS-MIE, Faculty of Medicine, University of Buenos Aires, UBA).

### Dynamic light scattering (DLS) measurements

DLS size distribution and hydrodynamic diameter measurements were performed at 25 °C with a Zeta sizer Nano-S apparatus (Malvern Instruments) using a low-volume quartz cuvette. OMVs samples in PBS were centrifuged at 13,000 rpm for 7 min before measurements. For each sample, the analysis consisted of 7 runs of 10 s carried out in duplicate. Size distributions and hydrodynamic diameters were calculated using the general-purpose distribution analysis model of the DTS v.7.11 software (Malvern Instruments Ltd.).

### Proteomic analysis

OMVs samples were loaded in SDS-PAGE in order to separate proteins from the lipid fraction. Proteins present in the samples were digested in gel with 100 ng Trypsin (Promega V5111) in 25 mM ammonium bicarbonate pH 8.0 ON at 37°C. Peptides were desalted using C18 zip tips (Merck Millipore) and eluted in 10 µl of H2O:ACN: FA 40:60:0.1%. The digests were analyzed by nanoLC-MS/MS in a nanoHPLC EASY-nLC 1000 (Thermo Scientific) coupled to a QExactive Mass Spectrometer. A 120 min gradient of H2O:ACN at a flow of 33 nl/min was used with a C18 2 mm Easy Spray column × 150 mm. Data Dependent MS2 method was used to fragment the top 12 peaks in each cycle. Raw data from mass spectrometry analysis was processed using the Proteome Discoverer 2.1.1.21 (Thermo Scientific) software for database searching with the SEQUEST search algorithm against the *B. suis* 1330 database. Precursor mass tolerance was set to 10 ppm and product ion tolerance to 0.05 Da. Protein hits were filtered for high confidence peptide matches with a maximum protein and peptide false discovery rate of 1% calculated by employing a reverse database strategy. A query result was only considered as significant if at least two tryptic peptides were detected with high confidence.

In order to perform Label Free Quantification (LFQ), samples were analyzed by triplicate. The area obtained for each protein was processed with the Perseus program (Max Planck Institute of Biochemistry, 1.5.5.3 version, available for free). Proteins that showed a fold change greater than 2 and a p-value below 0.05 were considered as differentially expressed.

### Animals

Female BALB/c mice (6–8 weeks old) were purchased from the National Academy of Medicine, Argentina, acclimated and randomly distributed into experimental groups. Animals were housed in a BSL3 animal facility (Operational Unit, Center for Biological Containment, National Administration of Laboratories and Health Institutes “Dr. Carlos G. Malbrán,” Argentina.), and received water and food *ad libitum*. Experiments in mice were approved by the animal care and use committee of the Faculty of Pharmacy and Biochemistry, UBA (REDEC-2022-1299-E-UBA-DCT-FFYB).

### Immunization

#### Experiment I

Mice were distributed into three experimental groups (n=5/group) and were immunized at 0 and 30 days by intramuscular (i.m.) injection of 20 µg of *B. suis* wt OMVs (wt OMVs), 20 µg of wt OMVs plus 10 µg CpG-ODN 1826 adjuvant (Invivogen) or sterile physiological saline solution (SS). At different time points after immunization, blood samples were collected for antigen-specific antibody determination and the animals were subjected to protection assays (see below).

#### Experiment II

Five animals per group were i.m. immunized with 20 µg wt OMVs, 20 µg *B. suis* Δ*mapB* OMVs (Δ*mapB* OMVs) or SS at 0 and 30 days. At different time points after immunization, blood samples were obtained for antibody quantification and spleens were harvested to assess cell-mediated immunity. Additionally, a subset of animals from each group was subjected to protection assays.

### Determination of antibody response

OMVs-specific IgG and IgA levels were determined in serum samples collected at 23 and 42 days after the initial mice immunization. Mucosal-specific IgA was measured in saliva, fecal extracts, and bronchoalveolar fluid (BALF). Specific antibodies were determined using an indirect homologous ELISA. Polystyrene plates (Corning Incorporated, New York, USA) were coated with 0.5 µg/well of either wt OMVs or Δ*mapB* OMVs in PBS and incubated overnight at 4°C. The plates were then washed three times with PBS containing 0.05% Tween-20 (PBS-T), followed by blocking with PBS containing 3% skim milk for 2 hours at 37°C. After blocking, plates were incubated for 2 hours at 37°C with the appropriate dilutions of the samples in blocking buffer. Samples from animals immunized with wt OMVs were tested on plates coated with wt OMVs, while samples from animals immunized with Δ*mapB* OMVs were tested on plates coated with Δ*mapB* OMVs. Following sample incubation, plates were washed three times with PBS-T and then incubated for 2 hours at 37°C with the appropriate dilutions of isotype-specific goat anti-mouse horseradish peroxidase (HRP) conjugates (Sigma Aldrich, Missouri, USA; Jackson ImmunoResearch, Pennsylvania, USA). After washing with PBS-T, the reaction was developed by adding TMB substrate (BD TMB Substrate Reagent Set, BD Bioscience, San Diego, USA). After 20 minutes of incubation at room temperature, the reaction was stopped by adding 2N H_2_SO_4_, and the optical density (OD) was measured at 450 nm using a microplate reader (Multiskan). ELISA assay cut-off values were calculated as the mean OD plus 3 standard deviations (SD) from sera of non-immunized mice. Serum titers were determined as the reciprocal of the highest dilution with an OD above the cut-off value. In some figures, to facilitate comparison between groups, the OD of antigen specific -IgG at a single dilution is plotted, as indicated in the figure legend.

To assess whether *B. suis* OMV antigens can induce an antibody immune response in the natural host during natural infection, an indirect ELISA assay was performed as described above. Sera from 14 healthy and 14 diseased pigs (provided by the Brucellosis Department, DILAB, SENASA) were tested at appropriate dilution in triplicate as previously described (42). In this case, HRP-conjugated goat anti-swine IgG at the appropriate dilution (Jackson ImmunoResearch) was used as the secondary antibody.

### Heterologous ELISA

To evaluate the cross-reactivity of immune sera from each vaccination group, we performed heterologous indirect ELISA as previously described for homologous assays. Immune sera from each experimental group were tested against the antigen used for vaccination in the other group. To standardize comparisons between assays with different antigens, OD values were converted into Standard Deviation Scores (SDS) using the following equation:


SDS=(ODs−ODni)/SDni

ODs: OD of sample

ODni: mean OD of non-immune sera

SDni: SD of non-immune

### Anti-lipopolysaccharide antibodies

To evaluate anti-LPS response elicited by each vaccine, sera from immunized mice were tested by indirect ELISA, using plates coated with 0.1 µg/mL of *Brucella abortus* LPS (provided by Ignacio Moriyón, University of Navarra, Pamplona, Spain), as described previously (43).

### Neutralization assay

The capacity of antibodies to neutralize bacterial adhesion and invasion of epithelial cells was evaluated as described ​ (44)​. Briefly, a suspension of *B. suis* 1330 was incubated with a 1/10 dilution of decomplemented sera from immunized mice and control groups for 1 hour at 37°C with gentle agitation. After incubation, the bacterial suspension was used to infect a confluent monolayer of A549 human lung epithelial cells (ATCC CCL-185) in 96-well plates (5 x 10^4^ cells/well) at a multiplicity of infection (MOI) of 100 for 1 hour at 37°C in 5% CO_2_. The cells were then washed with sterile PBS, and the total bacteria associated with the cells were determined by lysis with 0.2% Triton X-100, followed by plating serial dilutions. To determine bacterial invasion, the number of intracellular viable bacteria was quantified by incubating the infected monolayers with 100 μg/ml gentamicin (Sigma Aldrich) to eliminate extracellular. Following this treatment, the cells were washed and lysed. The resulting lysates were serially diluted, plated onto TSA, and incubated at 37°C for 5 days. After incubation, colony-forming units (CFU) were counted to quantify bacterial viability. The number of adherent bacteria was calculated as the difference between the total bacteria associated with the cells and the intracellular bacteria.

### Opsonophagocytosis assay

The opsonophagocytosis capacity of the antibodies was assessed as described (44). Briefly, *B. suis* was resuspended in a 1:10 dilution of pooled sera from immunized mice of each experimental group. After 1 hour of incubation at 37°C, the bacterial suspension was used to infect murine macrophages (RAW 264.7 cell line) at MOI of 100 in 96-well plates (5 x 10^4^ cells/well) for 1 hour at 37°C (time 0 p.i.). After incubation, the cells were washed and lysed to determine the number of bacteria adhered and internalized as described above.

### Characterization of cellular immune response

Antigen Presenting Cells (APCs): TIB-208™ cells, a B cell lymphoma, (1 x 10^6^ cells/ml) were cultured overnight in complete RPMI 1640 (10% fetal bovine serum, 0.05 mM 2-mercaptoethanol, 1 mM pyruvate, 2 mM L-glutamine, 100 U/ml penicillin and 100 μg/ml streptomycin) either alone or in presence of heat-killed *Brucella suis* (HKBs) (1x10^9^ CFU/ml) at 37 °C 5% CO_2_. After incubation, cells were treated with mitomycin (25 μg/ml) at 37°C for 45 min and washed twice with complete RPMI 1640 medium.

*In vitro* cytokine production: To evaluate whether vaccination with OMVs can induce a specific T cell immune response, splenocytes from immunized animals were harvested two weeks after the final immunization. These splenocytes (5 x 10^7^ cells/ml) were cultured in 48-well plates along with APCs (1 x 10^6^ cells/ml) pre-loaded with HKBs for 3 days at 37°C with 5% CO_2_. After 72 h of incubation, the supernatants were collected, and the levels of gamma interferon (IFN-γ), interleukin-17 (IL-17), and IL-5 were measured using commercial capture ELISA kits according to the manufacturer’s instructions (BD Biosciences).

### Protection experiments

Protection experiments were conducted as previously described by our group and others (25, 26, 28, 29, 44, 45). Two weeks after the final immunization, mice were challenged either intraperitoneally (i.p.) with 1x10^4^ CFU or intratracheally (i.t.) with 5x10^4^ CFU of *B. suis* 1330. Two weeks post-challenge, mice were euthanized, and spleens (i.p. challenge) or both lungs and spleens (i.t. challenge) were aseptically collected. The organs were individually homogenized in 2 ml of sterile PBS, serially diluted ten-fold, and each dilution was plated onto two TSA plates. Plates were incubated at 37°C for 3-5 days. CFU were counted and expressed as log_10_ CFU per organ. Results for each organ were reported as the mean CFU/organ ± standard deviation (SD) for each group. The level of protection was calculated as the difference in mean log CFU between the vaccinated group and the SS control group.

In the absence of an approved vaccine for *B. suis* infections, the attenuated vaccine strain *B. abortus* S19 was included as a positive control in these protection experiments. A group of mice received an i.p. injection of *B. abortus* S19 (1x10^4^ CFU/mouse) one month prior to the challenge, using the dose previously described (28, 29, 45).

Protection was defined as the log10 of reduction in CFU in the spleen (i.p. and i.t. challenges) and lung (i.t. challenge) of immunized mice compared to the negative control group (SS).

### Statistical analysis

Data were tested for Shapiro-Wilk Normality and Bartlett variance homoscedasticity. For experiments with more than two groups, results were analyzed using analysis of variances (ANOVA) or Kruskal Wallis test and comparison against the control group was made with the Dunnet, Tuke’y or Dunn post-test, respectively. An unpaired *t-test* was used for comparison between two experimental groups. All statistical analyses were performed with the GraphPad Prism software (San Diego, CA).

## Results

### *B. suis* OMVs isolation and characterization

**Figure 1:**
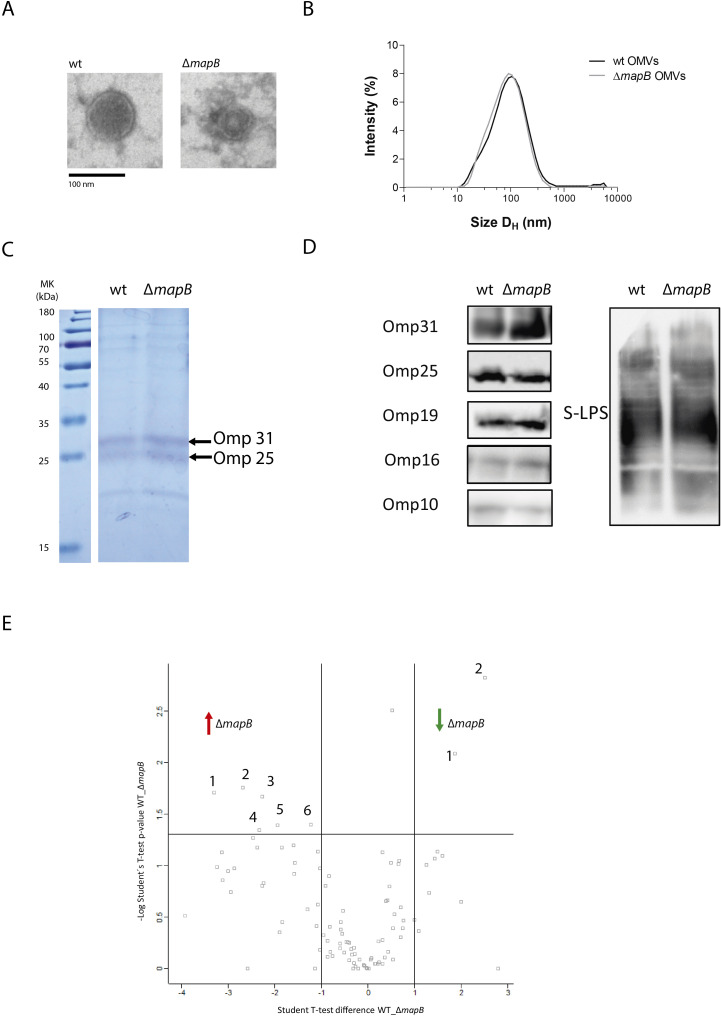
Characterization of *B. suis* 1330 and *B. suis* Δ*mapB* OMVs. wt OMVs and OMVs Δ*mapB* samples were analyzed by Electronic Microscopy **(A)** and Dynamic Light Scattering **(B)**, separated by SDS-PAGE 15% polyacrylamide gels **(C)** and transferred to a nitrocellulose membrane for immunoblot analysis with specific monoclonal antibodies anti-*Brucella* LPS, Omp31, Omp25, Omp19, Omp16 and Omp10 proteins **(D)**. Label Free Quantification (LFQ) proteomic assay was performed and samples were analyzed by triplicate. For each couple of samples, a volcano plot was constructed with –log p-value (-Log Student T-test p-value wt_Δ*mapB*) plotted on the y-axis versus Student T-test Difference wt_Δ*mapB* in the x axis **(E)**. Proteins with a fold change greater than 2 and a p-value below 0.05 were considered as differentially expressed.

We have previously reported the isolation of OMVs from *B. abortus* cultured in Gerhardt Wilson minimal medium (41). Due to its performance, the same method was applied in this work with minor modifications. OMVs fractions from *B. suis* wt and *B. suis* Δ*mapB* showed similar protein concentrations (approximately 8 mg protein/L culture) and spherical shapes, including a double membrane ​when observed by TEM (**Figure 1A**). Both OMVs samples showed a single particle population by DLS, which corresponded to a hydrodynamic diameter (HD) of 119 ± 9 and 105 ± 19 nm for the wt and Δ*mapB* OMVs, respectively (**Figure 1B**). The HD values agree with the size obtained by analytical TEM for both the wt and Δ*mapB* OMVs (122.8 ± 34.54 nm and 123.0 ± 43.95 nm, respectively).

Identifying proteins present in OMVs is crucial to understand their immunogenic properties. The SDS-PAGE analysis of wt and Δ*mapB* OMVs samples revealed a similar protein pattern (**Figure 1C**). Subsequent OMVs immunoblot analysis with specific monoclonal antibodies confirmed the presence of OM components such as *Brucella* S-LPS, Omp31, Omp25, Omp19, Omp16 and Omp10 proteins in both OMVs types (**Figure 1D**).

To assess the specific protein repertoire of the wt and Δ*mapB* vesicles a LFQ proteomic analysis was performed. A total of 94 and 95 hits were detected in the wt and Δ*mapB* OMVs, respectively, with 51 and 46 corresponding to membrane proteins. Proteomic data analysis revealed the presence of BLS, Omp16, Omp19, Omp31, Omp25, SOD, GroEL and bacterioferritin proteins in OMVs of both strains (**Supplementary Table S1**), all of which have been previously identified as promising *Brucella* vaccine candidates ​ (28–32)​. **Figure 1E** shows a Volcano plot highlighting differentially expressed peptides, with the detailed information summarized in **Table 1**. LFQ proteomic analysis revealed a reduction in the expression of Omp10 and an OmpA family protein in the Δ*mapB* OMVs (**Table 1**). Given the alterations previously observed in the *B. suis* Δ*mapB* cell envelope ​ (36), these results are particularly interesting since both OM proteins play a role in proper OM biogenesis. Conversely, six proteins were found to be increased in the Δ*mapB* OMVs, including one uncharacterized protein. Two of these proteins, an ABC transporter and a TRAP solute transporter, are periplasmic proteins predicted to be involved in amino acid uptake. The other three proteins were identified as a YaeC family lipoprotein, a periplasmic peptidyl-prolyl cis-trans isomerase with an N- terminal SurA domain and a cytosolic ribosomal protein. The increased presence of an ABC transporter and a TRAP solute transporter suggests, therefore, a compensatory strategy for nutrient uptake and homeostasis in the absence of MapB, potentially enhancing bacterial survival and virulence under stress. Moreover, the upregulation of the YaeC family lipoprotein likely supports membrane integrity and compensates for defects in outer membrane structure caused by the loss of MapB. In addition, the increased expression of a periplasmic peptidyl-prolyl cis-trans isomerase with a SurA domain highlights a possible upregulation of protein folding and assembly mechanisms to counteract disruptions in Δ*mapB* cell envelope. Taken together, these findings suggest that the Δ*mapB* strain activates compensatory pathways to maintain membrane function and bacterial adaptability.

**Table 1:** Label free quantification proteomic assay.

Reduced protein levels in Δ*mapB* OMVs		
ID	Description	Subcellular localization
1. A0A0H3G3E6	OmpA family protein BS1330_I1200 (motB)	OM
2. P0A3N9	Outer membrane lipoprotein Omp10	OM
Increased protein levels in Δ*mapB* OMVs		
1. A0A0H3G576	Uncharacterized protein. DUF922 domain-containing Zn-dependent protease	IM/C
2. A0A0H3G9A1	YaeC family lipoprotein nlpA lipoprotein	IM
3. A0A0H3G608	Peptidyl-prolyl cis-trans isomerase (surA domain)	IM
4. A0A0H3G440	TRAP transporter solute receptor TAXI family protein	P
5. Q8G3D4	Leu/Ile/Val-binding protein homolog. ABC transporter quorum sensing pathway	P
6. P59180	30S ribosomal protein S3 rpsC	IM/C

ID: Uniprot protein identification. Subcellular localization: determined by GnegmPloc online server (http://www.csbio.sjtu.edu.cn/bioinf/Gneg-multi/); OM, Outer Membrane; IM, Inner Membrane; C, Citosolic; P, Periplasmic.

**Figure 2:**
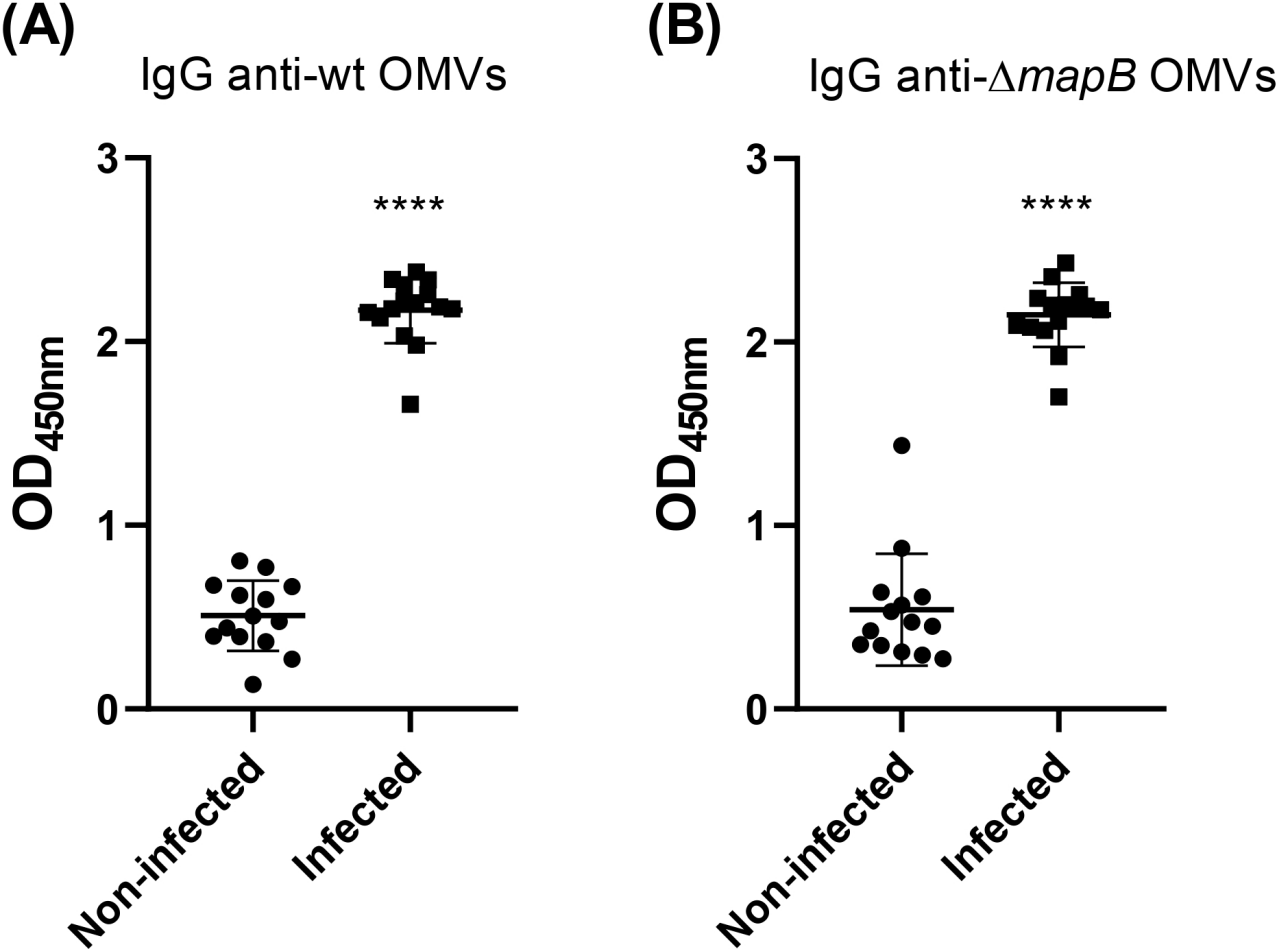
Specific IgG anti-wt OMVs **(A)** and anti-Δ*mapB* OMVs **(B)** from sera of sick (Infected) and healthy (Non-infected) natural pigs were determined by ELISA. Values express OD450 nm of sera 1/100 dilution. Data were analyzed by t-test followed by Mann Whitney post-test; ****p<0.0001 differences were calculated concerning the Non-Infected group. All results are representative of at least two independent experiments.

To determine whether *B. suis* OMVs antigens are immunogenic during infection of the natural host, we evaluated the presence of specific antibodies in sera from 14 healthy pigs and 14 pigs naturally infected with *B. suis*. IgG levels against both wt OMVs and Δ*mapB* OMVs were significantly higher in the sera of infected animals compared to healthy animals (**Figure 2**). These results indicate that antigens present in both OMVs induce an antibody response during natural infection.

### OMVs from *B. suis* are immunogenic

**Figure 3:**
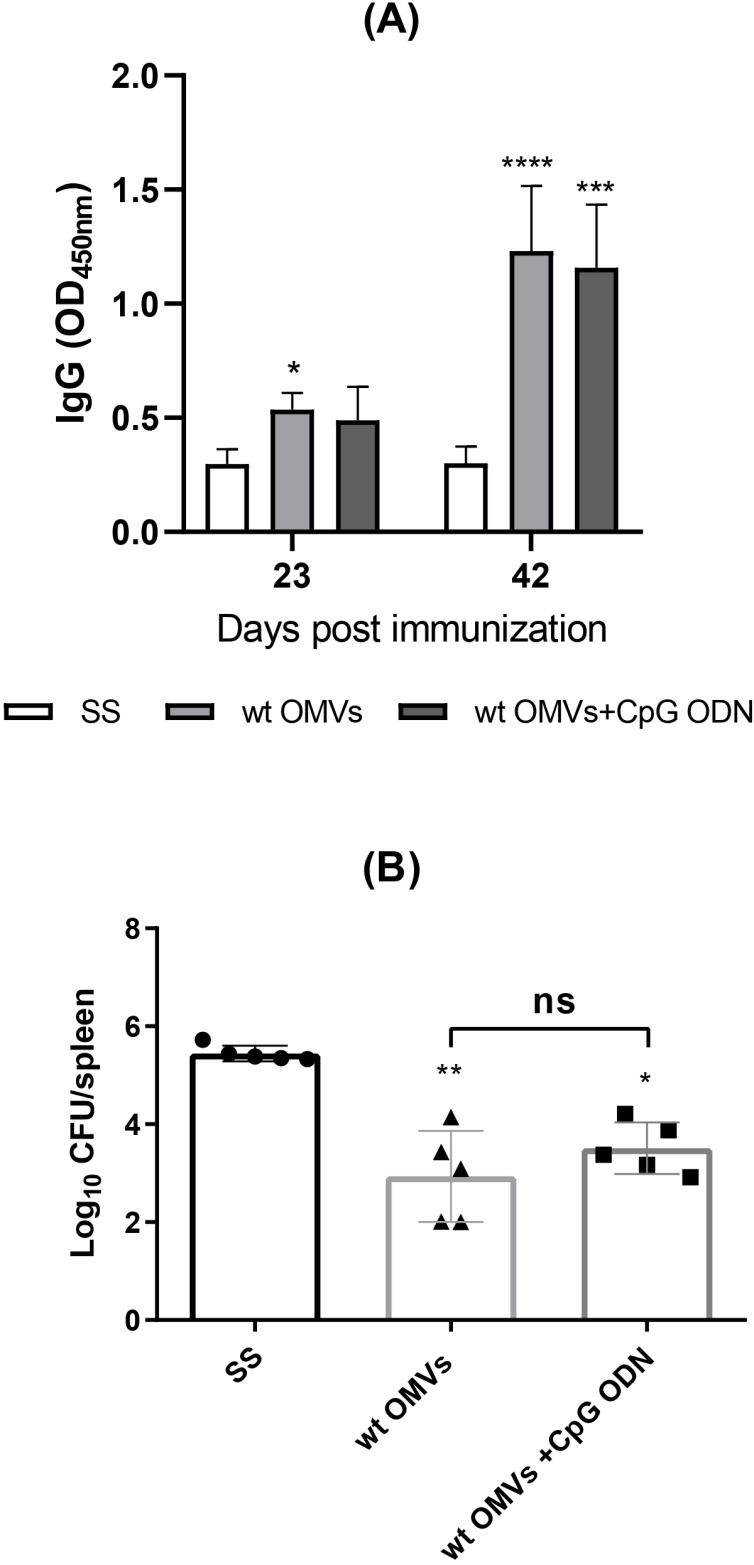
Immunization with wt OMVs induces a humoral immune response and reduces the splenic bacterial load. Mice were i.m. immunized with wt OMVs (20 µg), wt OMVs (20 µg) + CpG (10 µg), or saline solution (SS) at 0 and 30 days. At 23 and 42 days levels of specific anti-OMVs IgG antibodies in sera were measured by indirect ELISA **(A)**. Results are expressed as mean OD_450nm_ ± SD of sera 1/100 dilution. Immunized mice were challenged through the i.p. route with *B. suis* 1330. CFU were measured in spleens at 20 days post-challenge **(B)**. Data were analyzed by two-way ANOVA followed by Dunnett´s post-test, *p<0.05; **p<0.01; ***p<0.001; ****p<0.0001 differences were calculated concerning the control group (SS). All results are representative of at least two independent experiments.

As bacterial-derived products, OMVs inherently contain a range of molecules, including proteins and lipids, which activate the innate immune system. Prior studies have evaluated OMVs from pathogenic bacteria as vaccine platforms, demonstrating their ability to enhance immune responses, thus supporting their potential in vaccine development (20–26). To examine the immunogenic properties of OMVs isolated under our specific conditions, we assessed whether they could elicit significant immune responses when administered alone or if they required co-administration with adjuvants. For this purpose, mice were immunized by the i.m. route with wt OMVs alone or in combination with CpG-ODN adjuvant. wt OMVs immunization induced a specific humoral immune response both in the presence and absence of adjuvant (**Figure 3A**). Antibody levels increased significantly after the second immunization (30 days) in both experimental groups. However, no significant differences in serum IgG levels were observed between the wt OMVs and wt OMVs + CpG groups.

Following i.p. challenge with virulent *B. suis*, animals vaccinated with wt OMVs exhibited a significantly reduced splenic bacterial load compared to negative control (SS) (**Figure 3B**). No significant differences in the number of viable bacteria in the spleen were observed between animals immunized with OMVs alone or with OMVs + CpG. Taken together, these results demonstrate that OMVs obtained under our conditions are immunogenic when administered by the i.m. route, and partially protect mice from systemic *B. suis* infection.

### Δ*mapB* OMVs induces a stronger humoral immune response compared to OMVs

**Figure 4:**
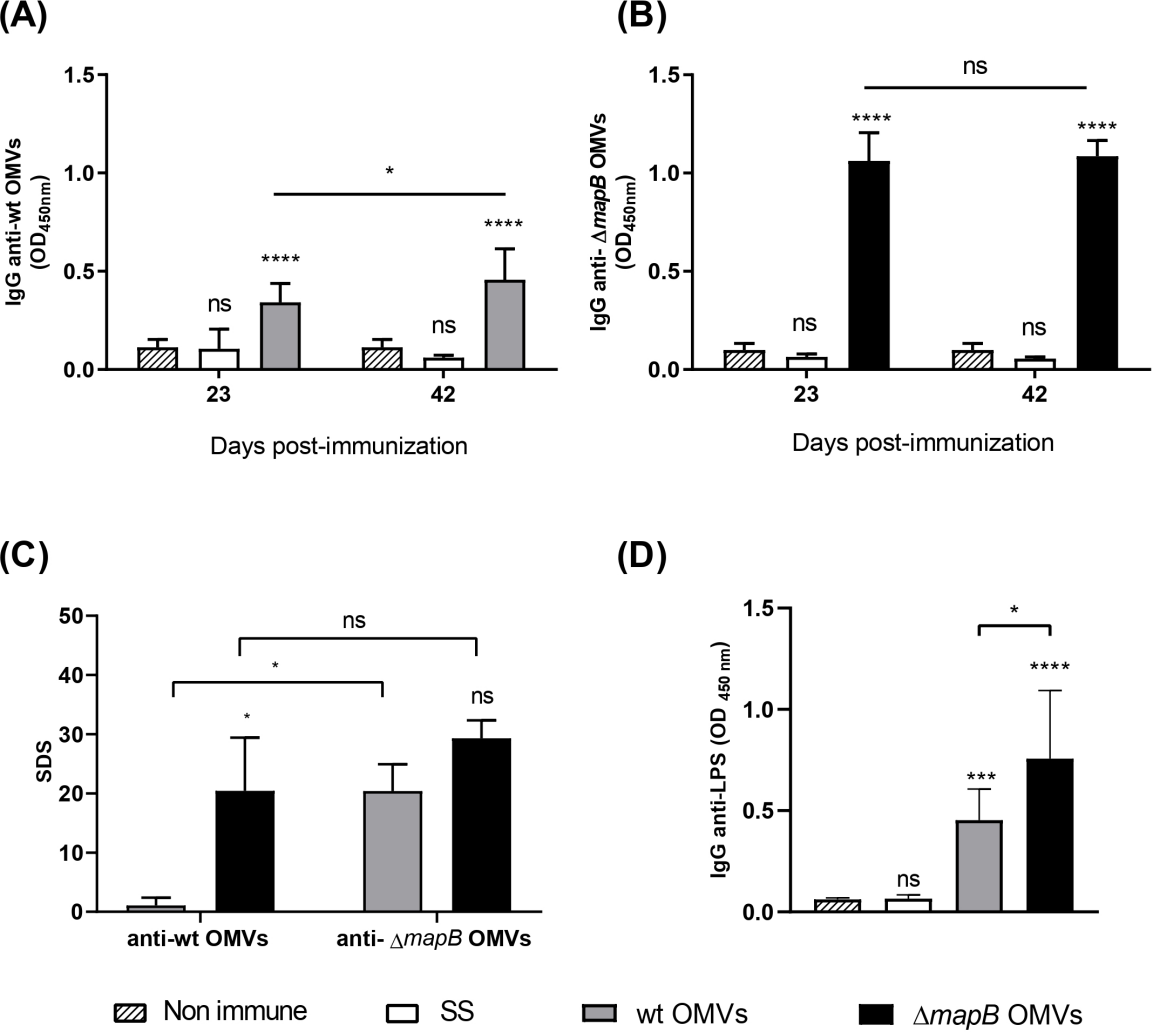
Antigen-specific serum IgG. Mice (n=5/group) were i.m. immunized with wt OMVs (20 µg), Δ*mapB* OMVs (20 µg), or saline solution (SS) at 0 and 30 days. Blood samples were collected at 23 and 42 days and levels of specific anti-wt OMVs or anti-Δ*mapB* OMVs IgG antibodies were measured by indirect homologous ELISA **(A, B)**. Data were analyzed by two-way ANOVA followed by the Dunnett test to compare against non-immune sera, and the Sidak test to compare the same group between times. Results are expressed as mean OD_450nm_ ± SD of sera 1/100 dilution. Sera cross-reactivity against heterologous OMVs was evaluated by indirect ELISA **(C)**. Results are expressed as standard deviation score (SDS), and Kruskal-Wallis statistical analysis followed by Dunn´s post-test was applied. Specific IgG anti- *Brucella* LPS were measured in serum collected at 42 days of wt OMVs and Δ*mapB* OMVs groups by indirect ELISA **(D)**. Results are expressed as mean OD450nm ± SD of sera 1/100 dilution. Data were analyzed by one-way ANOVA followed by the Tukey’s *post hoc* test to compare between groups.*p<0.05, **p< 0.01, ***p< 0.001, ****p<0.0001 vs. non-immune.

To evaluate the immunogenicity of *B. suis* Δ*mapB* OMVs and to compare the humoral immune responses elicited by both wt and Δ*mapB* OMVs, we assessed the production of specific antibodies in mice immunized via the i.m. route without adjuvant. Antibody levels were measured using an indirect ELISA against the respective immunogen (homologous ELISA). Both OMVs formulations induced specific IgG in serum (**Figures 4A, B**). Notably, the serum IgG titers were significantly higher in animals vaccinated with Δ*mapB* OMVs compared to those immunized with wt OMVs (median: 1600 for Δ*mapB* OMVs vs. median: 400 for wt OMVs).

To elucidate whether the higher antibody titer of animals immunized with Δ*mapB* OMVs was related to an increased ability of these vesicles to adhere to the ELISA plate, we assessed the cross-reactivity of sera from both vaccinated groups toward both wt and Δ*mapB* OMVs by heterologous indirect ELISA. Serum IgG reactivity induced by wt OMVs vaccination was higher against the Δ*mapB* OMVs than against the homologous antigen (wt OMVs) (**Figure 4C**). In contrast, serum antibodies from mice immunized with Δ*mapB* OMVs showed similar recognition toward both OMVs types (**Figure 4C**). These results demonstrate that there is no differential binding to the ELISA plate between both OMVs and suggest immunogenic differences between wt and Δ*mapB* OMVs instead.

To further characterize the immune response elicited by these vaccines, we also assessed anti-*Brucella* LPS antibodies in the sera of both vaccinated groups using an indirect ELISA. As shown in **Figure 4D**, vaccination with both OMVs types induced the production of anti-LPS antibodies, with significantly higher levels observed in the Δ*mapB* OMVs group. These findings reinforce the notion that Δ*mapB* OMVs elicit a stronger humoral immune response against *Brucella* antigens compared to wt OMVs, highlighting their enhanced immunogenic potential.

**Figure 5:**
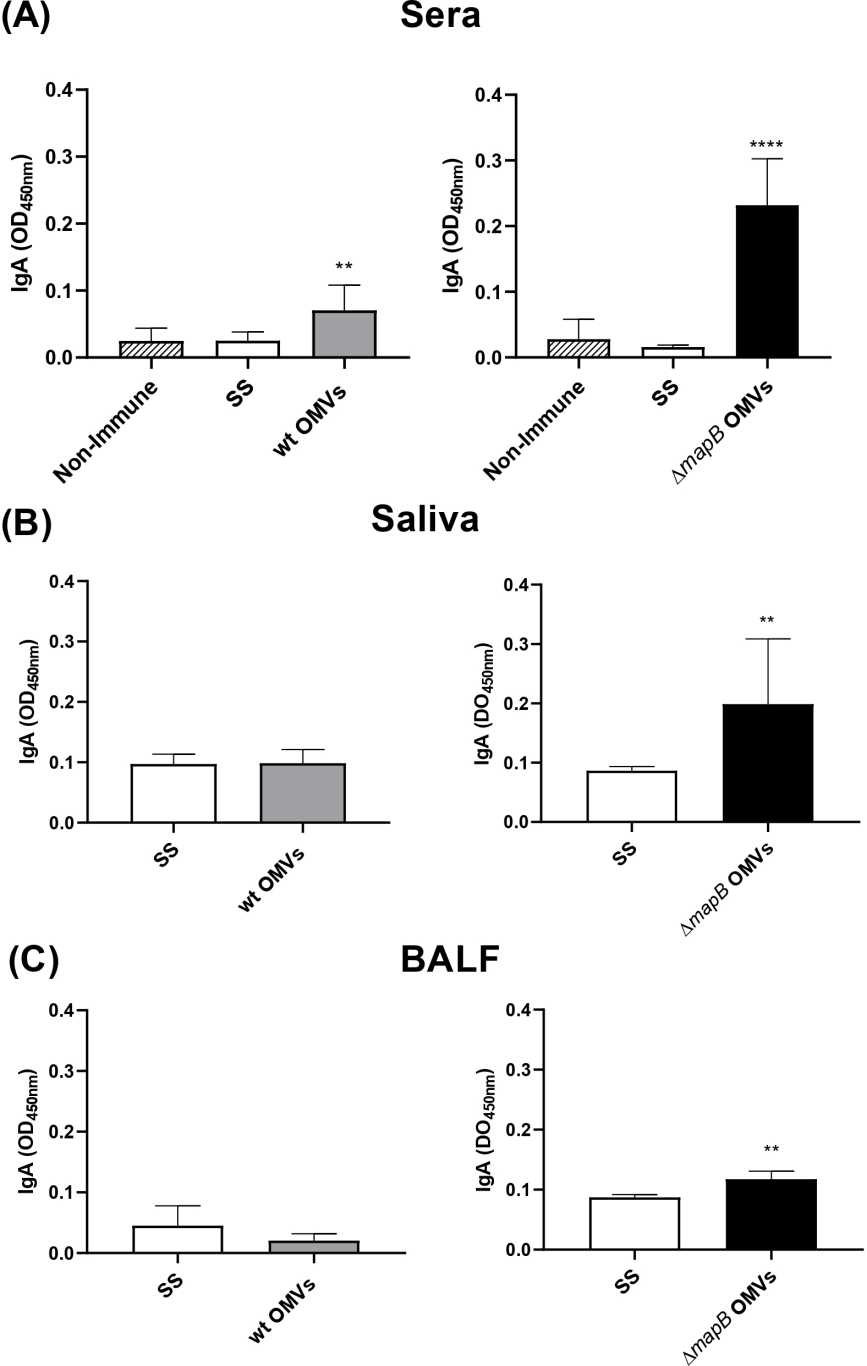
Specific serum and mucosal IgA. Mice were immunized with wt OMVs (20 μg), Δ*mapB* OMVs (20 μg), or saline solution (SS) at 0 and 30 days. Two weeks after the last immunization sera **(A)**, saliva **(B)**, and BALF **(C)** samples were obtained, and indirect ELISA measured specific IgA antibodies levels. Data were analyzed by one-way ANOVA followed by Dunnett´s test or by unpaired *t-test*. Data are expressed as mean OD_450nm_ ± SE. **p<0.01; ****p<0.0001 vs Non-Immune or SS.

In parallel, immunization with Δ*mapB* OMVs induced a greater increase in specific IgA titers in serum (median: 400) than wt OMVs (median: 100) (**Figure 5A**). Moreover, a slight increase of specific IgA levels in BALF and saliva was detected only in mice vaccinated with Δ*mapB* OMVs as compared with the control group (SS) (**Figures 5B, C**).

**Figure 6:**
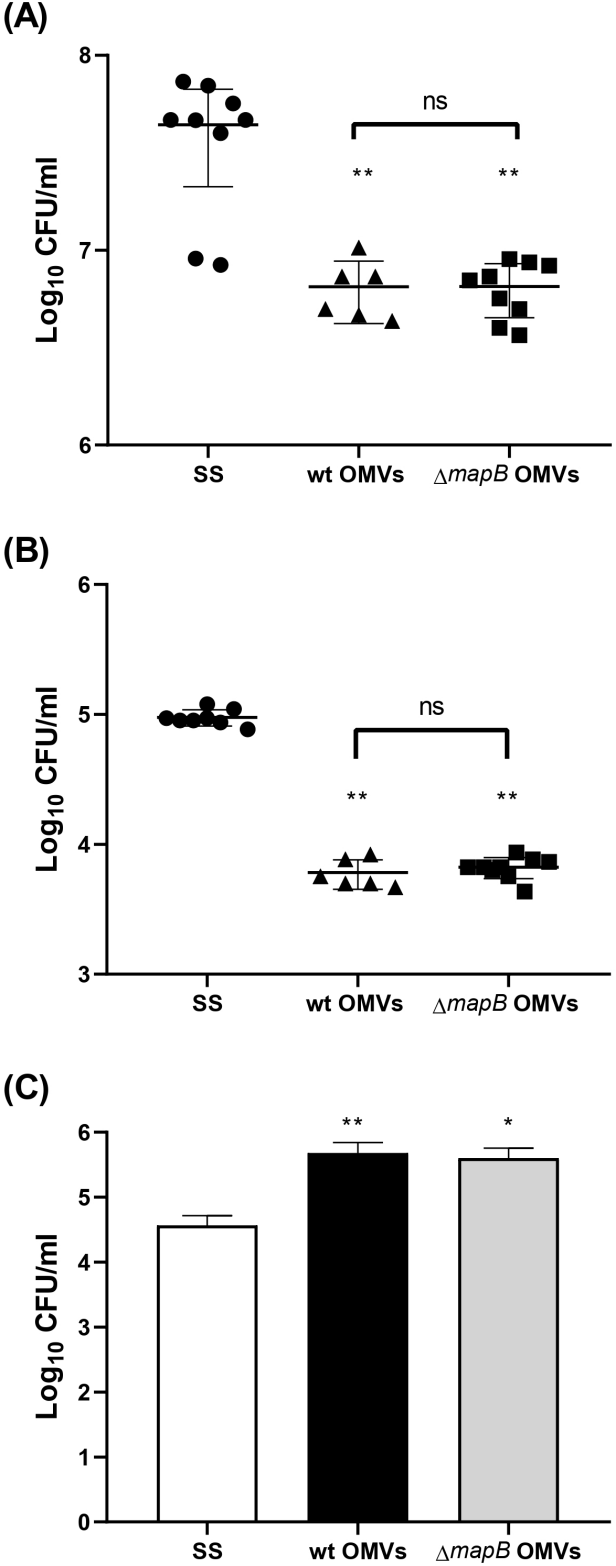
Serum antibodies’ functionality. Mice (n=5/group) were i.m. immunized with wt OMVs (20 µg), Δ*mapB* OMVs (20 µg), or saline solution (SS) at 0 and 30 days. Blood samples were collected at 23 and 42 days. Antibodies’ capacity to neutralize *B. suis* 1330 adhesion and invasion to A549 epithelial cells line was performed. Total bacteria adhered to the cell monolayer **(A)** and internalized bacteria **(B)** were determined. The ability of anti-OMVs antibodies to facilitate bacterial opsonophagocytosis was also determined. *B. suis* were preincubated with immune serum as described and added to macrophages for 1 h, then intracellular bacteria were determined for CFU counting **(C)**. Results are expressed as log CFU/ml. **p<0.01, *p<0.05 vs SS. Results are expressed as mean ± SD of duplicate measurements from three independent.

To assess the functionality of the specific serum antibodies induced by immunization, the neutralization capacity of these antibodies was evaluated. Serum antibodies from mice immunized with wt or Δ*mapB* OMVs significantly reduced bacterial adhesion and invasion to A549 epithelial cells monolayer compared to serum from the control group (**Figures 6A, B**). However, no significant differences were observed in this regard between sera from both OMVs-immunized groups. The ability of the serum specific antibodies to promote phagocytosis was also assessed. The uptake of *B. suis* by macrophages was significantly increased when bacteria were pre-incubated with sera from both OMVs-immunized groups as compared to sera from control mice (**Figure 6C**).

### Characterization of the cellular immune response

**Figure 7:**
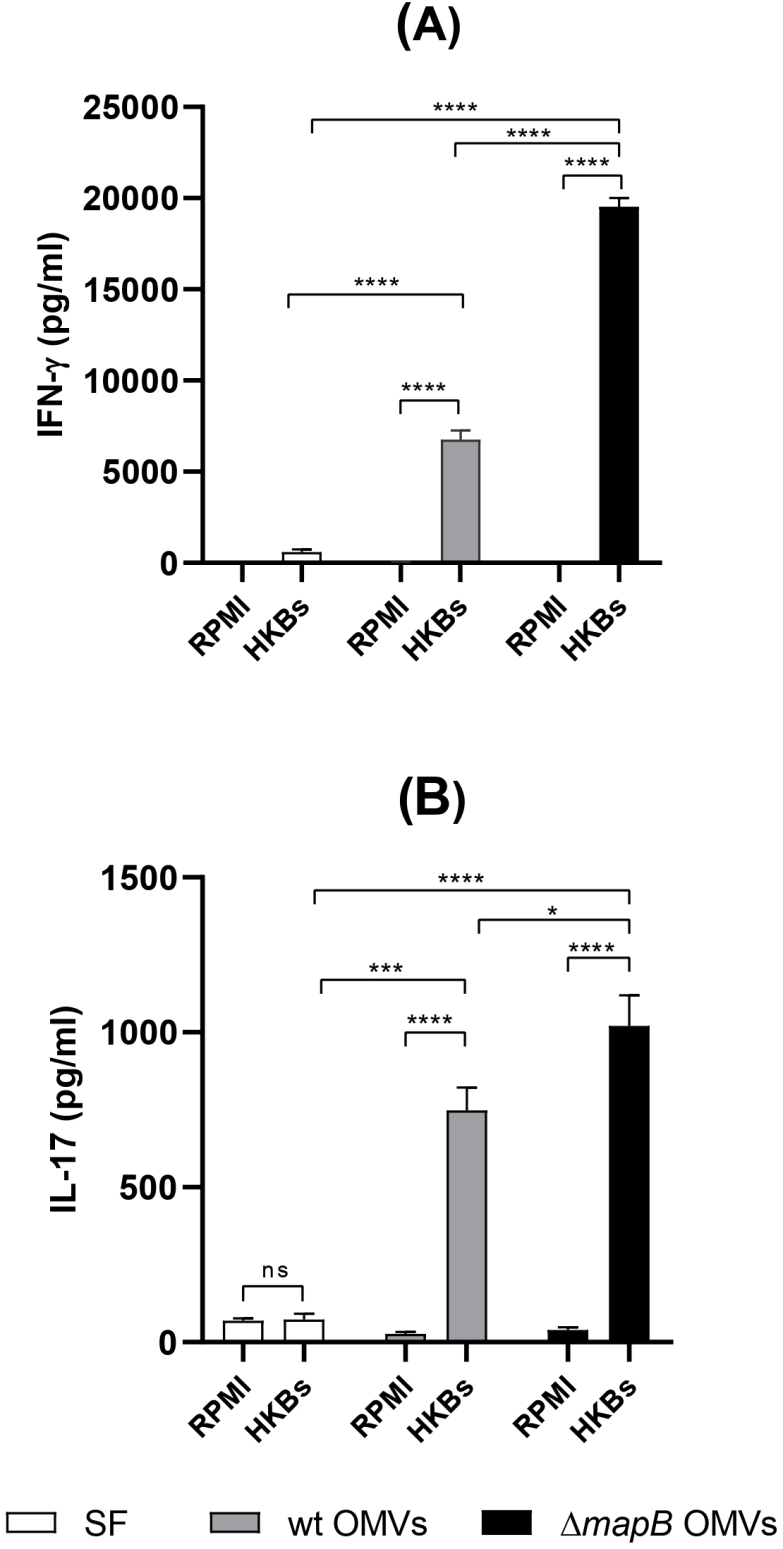
Cellular immune response. Two weeks after the last immunization, spleens from immunized mice (n=5/group) were collected and cell suspensions were cultured with APC pre-loaded with HKBs, or RPMI medium. At 72 h post-stimulation culture supernatants were harvested to measure levels of IL-17, and IFN-γ by ELISA **(A, B)**. Results were analyzed by two-way ANOVA followed by Dunnett’s multi-comparison test, and are expressed as the mean concentration ± SD of duplicate measurements from three independent experiments. (****p < 0.0001 vs RPMI 1640).

To characterize the T helper cellular immune response against *B. suis* antigens, spleen cells from immunized mice were stimulated *in vitro* with APCs pre-loaded with HKBs, and the cytokines IFN-γ, IL-17, and IL-5 were measured in the culture supernatants. Stimulation of spleen cells from the wt or Δ*mapB* OMVs groups significantly increased the secretion of IFN-γ and IL-17 compared to the control (SS) group (**Figures 7A, B**). Notably, spleen cells from the Δ*mapB* OMVs group secreted higher levels of IFN-γ and IL-17 than those from the wt OMV group (**Figures 7A, B**). Interestingly, IL-5 was undetectable in stimulated spleen cells from both wt and Δ*mapB* OMVs-vaccinated mice. These results suggest that vaccination with both wt and Δ*mapB* OMVs induces a mixed Th1 and Th17 response against *Brucella*.

### Immunization with Δ*mapB* OMVs induces systemic and mucosal protection

To evaluate the efficiency of OMV vaccination in protecting against *B. suis* infection, immunized animals were challenged via the i.p. route with virulent *B. suis*. Immunizations with both OMVs reduced the spleen bacterial load after *Brucella* infection compared to the control group (**Table 2**). Notably, vaccination with Δ*mapB* OMVs conferred significantly greater protection, reducing the bacterial load by 2.113 log compared to 1.673 log observed with wt OMVs (p<0.05). For reference, the S19 vaccine strain, included as a positive control, reduced the bacterial load by 1.474 log (p<0.05 compared to Δ*mapB* OMVs). Although this strain is not an approved vaccine for preventing *B. suis* infection, a recent study has demonstrated its safety and immunogenicity in pregnant pigs (46), emphasizing its potential as a candidate vaccine against *B. suis* in swine.

**Table 2:** Protection from virulent *B. suis* challenge in mice.

Intraperitoneal challenge			
	Experimental group	log CFU/organ (mean ± SD)	Protection level (log)
Spleen	SS	5.421 ± 0.093^a^	0.00
	wt OMVs	3.748 ± 0.180^b^	1.673
	Δ*mapB* OMVs	3.308 ± 0.039^c^	2.113
	*B. abortus* S19	3.947 ± 0.420^b^	1.474
Intratracheal challenge			
Spleen	SS	5.281 ± 0.185^a^	0.00
	wt OMVs	4.108 ± 0.165^b^	1.173
	Δ*mapB* OMVs	4.159 ± 0.104^b^	1.122
	*B. abortus* S19	4.058 ± 0.489^b^	1.223
Lung	SS	5.493 ± 0.129^a^	0.00
	wt OMVs	4.847 ± 0.097^b^	0.646
	Δ*mapB* OMVs	4.822 ± 0.135^b^	0.671
	*B. abortus* S19	3.911 ± 0.187^c^	1.582

BALB/c mice from wt OMVs, Δ*mapB* OMVs *B. abortus* S19 and saline groups (SS) were challenged through the i.p. and i.t. route with *B. suis* 1330. CFU were measured in spleens and lungs at 20 days post-infection. Values are expressed as mean CFU/organ ± SD for each vaccine group of duplicate measurements from two independent experiments. Significant differences between groups were analyzed by one-way ANOVA followed by Tukey’s test statistical analysis and are indicated by lowercase letters, at p <0.05.

To assess whether the immune response triggered by i.m. immunization with these OMVs was able to protect against mucosal infection, immunized mice were challenged via i.t. route with *B. suis*. Vaccination with both OMVs significantly reduced *Brucella* spleen load, showing protection levels like those observed in the S19 vaccine group (**Table 2**). Vaccination with both OMVs also significantly reduced (0.646 and 0.671 respectively) the lung *B. suis* burden compared to the control group (SS), albeit to lower levels than the S19 vaccine (1.582) (**Table 2**).

Together these results suggest that immunization with both OMVs protects against i.p. challenge, and partially against i.t. challenge with *B. suis*.

## Discussion

*B. suis* is the etiological agent of swine brucellosis, a widely distributed zoonotic disease for which there are still no approved vaccines. OMVs have garnered significant attention as potential vaccine candidates due to their ability to stimulate robust immune responses. These vesicles, naturally released by many Gram-negative bacteria, contain a variety of surface-exposed antigens and PAMPs, which can activate both innate and adaptive immune systems. The presence of multiple immunogenic components, such as LPS, Omps, and other molecular motifs, allows OMVs to act as a powerful platform for immunization, providing broad protection against a range of bacterial pathogens. This inherent diversity in antigenic profiles, combined with their ability to mimic bacterial infections without the risk of virulence, makes OMVs an attractive option for developing vaccines, especially in the context of pathogens that lack effective vaccine candidates (17–24). Recently, we demonstrated that *B. suis* MapB protein is essential for OM stability and is involved in cell division ​ (36)​. In this work, we characterized OMVs from *B. suis* wt and *B. suis* Δ*mapB* and, for the first time, evaluated the potential of these vesicles as an acellular vaccine against *Brucella* infection.

To obtain better yields of OMVs, we employed a production strategy previously described in our laboratory, with minor modifications (41)​. OMVs obtained from both strains under these conditions showed spherical shapes with bilayer-double membranes and ranged in size from 90 to 100 nm. These OMVs have a similar size to previously reported OMVs from *B. abortus* and *B. melitensis* (25, 26, 41), albeit slightly larger than that of OMVs isolated from *B. suis* grown on solid medium (47 nm) ​ (27)​. It is worth noting that the characteristics and composition of OMVs can vary depending on the methods used for isolation and purification, culture medium, conservation techniques, and bacterial stress conditions ​ (26, 27)​. Therefore, the variations in growth conditions across these studies could account for the differences in OMVs size.

Proteomic analysis of OMVs isolated from the wt and Δ*mapB* strains revealed the presence of 94 and 95 proteins, respectively, with similar subcellular localization in both strains. We previously demonstrated that MapB deficiency in the mutant strain did not lead to an extensive alteration of Omp abundance in total membranes, but rather caused a reduction in the relative amounts of a subset of proteins, including Omps ​ (36)​. Notably, under the assayed conditions, the proteomic approach showed a significant reduction in the abundance of Omp10 and a protein from the OmpA family (BR1204) in Δ*mapB* OMVs. Previous studies have indicated that deletion of the Omp10 gene in *B. abortus* affects its survival *in vivo* but does not impact on its membrane properties ​ (47)​. Regarding OmpA, it is interesting to note that the OmpA homologue from the gammaproteobacterium *Pseudomonas aeruginosa* possesses a conserved domain that anchors the OM to the peptidoglycan, playing a crucial role in OMVs biogenesis (48)​. Furthermore, down-regulation of the OmpA protein has been found to enhance OMVs production (48)​. Therefore, OmpA deficiency could potentially contribute to the instability of OM in the Δ*mapB* mutant strain of *B. suis*.

Interestingly, SurA, a periplasmic peptidyl-prolyl cis-trans isomerase protein, is included among the increased proteins in Δ*mapB* OMVs. SurA is both a folding catalyst and a chaperone in β-barrel Omps biogenesis and as such plays a key role in cell envelope homeostasis (45, 49, 50)​. In addition, SurA is also required for full virulence of uropathogenic *Escherichia coli, Shigella* and *Salmonella* spp (49). A previous study showed that immunization with recombinant SurA from *B. abortus* induces a Th1/Th2 immune response and confers partial protection against *B. abortus* challenge (45); so, the increased levels of this protein may confer Δ*mapB* OMVs an advantage as immunogen.

Among the proteins shared by OMVs of wt and Δ*mapB* strains, we identified BLS, Omp16, Omp19, Omp31, Omp25, SOD, GroEL and bacterioferritin, which have been previously recognized as either virulence factors or protective immunogens against *Brucella* (29–32, 51). The presence in the OMVs of the mentioned Omps was confirmed by Western Blot. Supporting our findings, a study by Boigegrain et al. in 2004 showed the presence of Omp31 and Omp25 proteins in OMVs from *B. suis* (52). Furthermore, in a recent study, Ruiz Palma et al. identified immunogenic proteins, such as Omp16, Omp25, Omp31, and SodC in the proteome of OMVs from *B. suis* 1330 (26). Omp25 is required for intracellular survival of *Brucella* in macrophages (53). In addition, *B. suis* Omp25 inhibits TNFα production, a key cytokine in bacterium control, by inducing specific microRNA expression in macrophages (30, 53). Omp31 is involved in *B. melitensis* membrane stability and the inhibition of TNFα-induced macrophage apoptosis (54). Of note, it was reported that Omp16 and Omp19 activate dendritic cells and induce immune protection similar to that elicited by the *B. abortus* S19 vaccine (28, 29). In this study we showed that OMVs from both strains also contain SOD and GroEL. These proteins play a role in bacterial resistance mechanisms to macrophagic microbicide activity. While previous studies have shown that immunization with GroEL does not generate a protective response against *Brucella* (51, 55), vaccination with SOD has demonstrated effective immune protection in the natural host (51).

The presence of immune-protective proteins in OMVs from *B. suis* makes them promising candidates for an acellular vaccine. Importantly, both wt and Δ*mapB* OMVs were recognized by serum antibodies from infected pigs, confirming their immunogenicity in the natural host.

The present study showed that, similarly to OMVs from *B. abortus* and *B. melitensis* (25, 26),​ OMVs isolated from *B. suis* can stimulate the immune response and confer protection in mice. Vaccination with wt OMVs alone generated serum specific antibodies and significantly reduced *B. suis* burden in the spleen of infected mice. Notably, the addition of CpG ODN adjuvant to the vaccine formulation did not enhance antibody levels or improve the protection achieved by OMVs alone. These findings indicate that OMVs from *B. suis* are immunogenic and protective at the given dose, without requiring additional adjuvants.

The absence of MapB in *B. suis* causes membrane instability, which may confer distinct immunogenic characteristics to the OMVs released by the mutant strain. In this study, we investigated the humoral immune response induced by vaccination with OMVs from both wt and Δ*mapB* strains. Immunization with Δ*mapB* OMVs produced higher serum levels of specific IgG and IgA compared to immunization with wt OMVs. This difference was not due to a methodological artifact, as sera from animals immunized with Δ*mapB* OMVs recognized both wt and Δ*mapB* OMVs similarly. However, sera from animals vaccinated with wt OMVs exhibited stronger recognition of mutant OMVs. Furthermore, Δ*mapB* OMVs induced significantly higher levels of *Brucella* LPS-specific antibodies compared to wt OMVs, highlighting their enhanced capacity to elicit humoral immune responses against this key *Brucella* antigen.

These results confirm the immunological distinctions between the two types of OMVs, which could be attributed to differences in protein abundance, as indicated by proteomic analysis, or to variations in the exposure or accessibility of certain antigenic epitopes within the structure of Δ*mapB* OMVs. The MapB homolog, TamB, has been implicated in maintaining cell envelope homeostasis, as demonstrated in our previous work (36) and in other bacterial genera (56–58). As a member of the AsmA protein family, TamB has been proposed to mediate the transport of phospholipids from the inner membrane (IM) to the OM in diderm bacteria. Supporting this, studies in *E. coli* have shown differential phospholipid composition between the IM and OM (34, 59). A membrane with altered lipid composition may affect its organization and fluidity, subsequently modifying the accessibility of surface antigens. These changes could explain the increased immunogenicity observed in OMVs derived from the Δ*mapB* mutant strain. Further research will be required to confirm the contribution of MapB to the phospholipid composition of *Brucella* membranes and to better understand how these changes influence the immunogenicity and structural properties of OMVs from the mutant strain.

Importantly, serum from both the wt and Δ*mapB* OMVs groups was able to neutralize bacterial adhesion and invasion of lung epithelial cells, and both mediated opsonophagocytosis by macrophages. Interestingly, immunization with Δ*mapB* OMVs elicited a slight increase in specific IgA levels in saliva and BALF, suggesting that parenteral immunization with these vesicles may promote a limited but specific mucosal immune response.

In our study, OMVs from *B. suis* strains conferred protection against i.p. *B. suis* infection. This result is consistent with previous studies conducted by Avila Calderon et al. (25) and Araiza Villanueva et al. (26), which demonstrated that OMVs from *B. melitensis* and *B. abortus* confer comparable protection to vaccine strains against parenteral challenge with *B. melitensis* and *B. abortus*, respectively. Interestingly, immunization with Δ*mapB* OMVs achieved higher levels of protection compared to immunization with wt OMVs, suggesting that the enhanced protective effect is linked to the more robust immune response elicited by Δ*mapB* OMVs.

Since *Brucella* primarily invades hosts through the mucosal routes, the development of a vaccine capable of inducing a protective mucosal response is highly desirable. Although it is widely recognized that parenteral immunization typically fails to generate protective mucosal immune responses, recent studies have revealed that OMVs derived from *Bordetella pertussis* and *Bordetella parapertussis* can effectively protect against mucosal infection even when administered through non-mucosal routes (22, 60). Notably, i.m. vaccination with wt OMVs and Δ*mapB* OMVs provided partial protection against respiratory challenge with *B. suis*, leading to reduced splenic and lung CFU counts. To the best of our knowledge, this study represents the first evidence that an acellular vaccine based on OMVs from *Brucella* can confer partial protection against mucosal challenge.

While antibodies targeting the O-antigen of LPS can provide some degree of protection in certain host species, the primary defense against virulent *Brucella* infection relies on cell-mediated immunity (61). Since *Brucella* species are facultative intracellular pathogens that reside primarily in macrophages, cellular immune responses involving IFN-γ-producing CD4+ T cells are considered critical for protective immunity (50, 51). IFN-γ is often the most essential effector cytokine for activating macrophages, enhancing their ability to eliminate and inhibit replication of intracellular microbial pathogens (50, 51).

**Figure 8:**
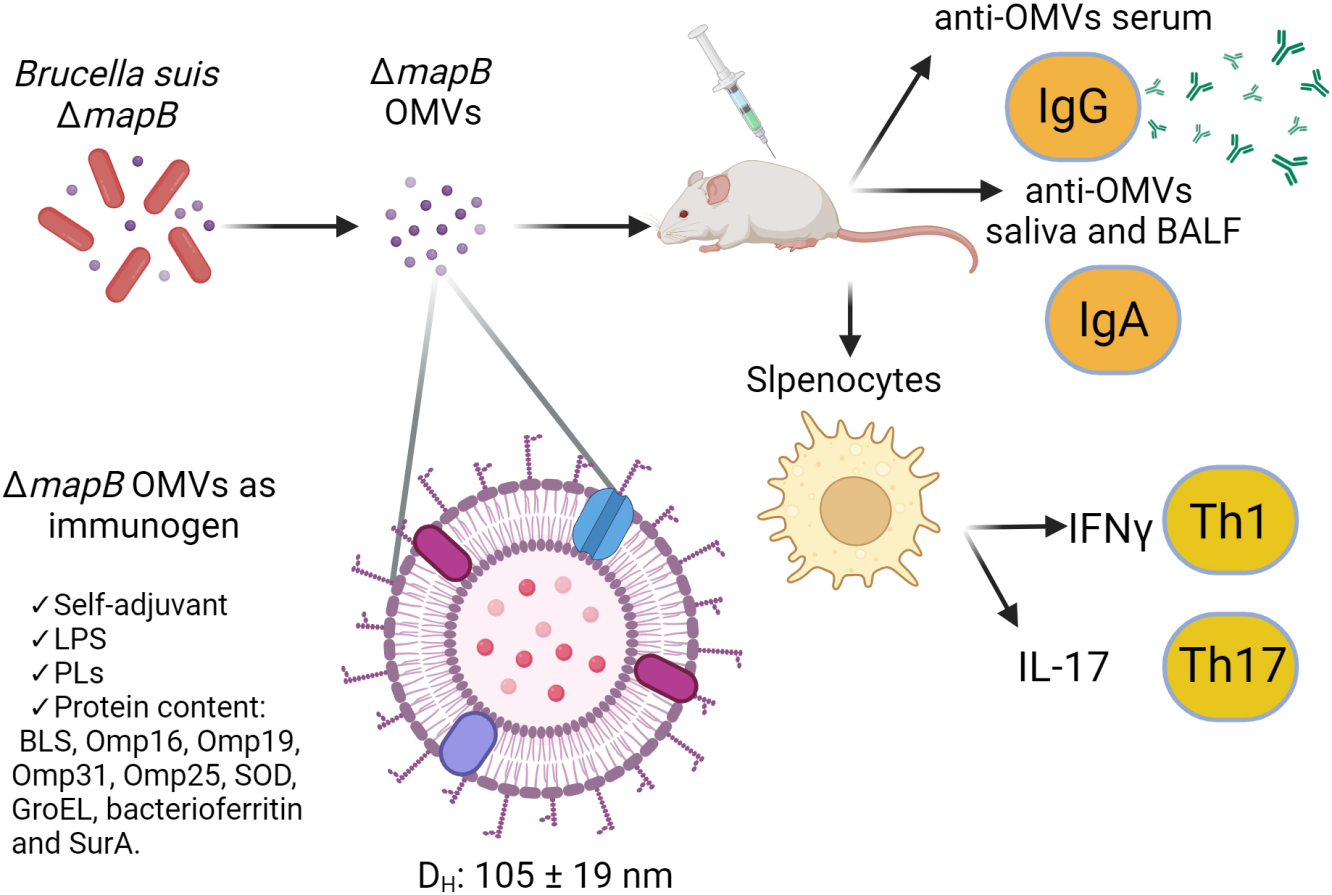
Model of the immune response elicited by Δ*mapB* OMVs from *Brucella suis* as an immunogen. This model illustrates the immune response induced by OMVs derived from a *Brucella suis* Δ*mapB* mutant. The OMVs, are spherical with a hydrodynamic diameter (D_h_) ranging from 105 to 119 nm. These OMVs contain key immunomodulatory components including LPS, phospholipids (PLs), and immunogenic proteins (BLS, Omp16, Omp19, Omp31, Omp25, SOD, GroEL, bacterioferritin, and SurA, among others), and act as a self-adjuvanting immunogen. Upon immunization in a murine model, OMVs trigger the production of anti-OMVs IgG in serum and anti-OMVs IgA in saliva and bronchoalveolar lavage fluid (BALF). Additionally, splenic cells from immunized mice were stimulated, leading to a Th1 immune response characterized by IFN-γ production and a Th17 response marked by IL-17 secretion.

In the present work, we demonstrate that immunization with wt or Δ*mapB* OMVs induces specific T cells capable of secreting high levels of IFN-γ and moderate levels of IL-17 in response to antigen stimulation. In particular, immunization with OMVs Δ*mapB* produced higher levels of IFN-γ than immunization with wt OMVs, which may explain the higher levels of protection against systemic infection conferred by vaccination with vesicles from the mutant strain. Interestingly, Hanot Mambres et al. (62) demonstrated that the nature of the protective memory response is closely dependent on the pathway of *Brucella* infection and highlighted the necessity of IFN-γ and IL-17RA for controlling mucosal infection by *Brucella melitensis* (62). This background may explain the protection conferred by vaccination with both OMVs against mucosal challenge with *B. suis*. Nevertheless, further studies are needed to characterize the profile of antigen-specific T cells induced by OMV vaccination at mucosal sites. As depicted in **Figure 8**, immunization with Δ*mapB* OMVs induced both mucosal and systemic immune responses, as evidenced by the production of anti-OMV IgG and IgA, as well as the activation of Th1 and Th17 cells, marked by IFN-γ and IL-17 secretion, respectively.

In this study, immunization with OMVs from both *B. suis* strains provided a protective immune response against virulent *B. suis* infection at both mucosal and systemic levels. However, we propose the use of Δ*mapB* OMVs as a preferred vaccine candidate for the following reasons: 1) immunization with these OMVs showed superior protective efficacy, compared to wt OMVs in a parenteral challenge, 2) immunization with Δ*mapB* OMVs induced a more robust humoral and cellular immune response compared to immunization with wt OMVs, and 3) the attenuated phenotype of the mutant strain improves the safety profile of the vaccine production process.

Overall, our study is the first to demonstrate *B. suis* OMVs immunogenicity and protective efficacy conferred against both systemic and respiratory challenges by virulent *B. suis*.

This work provides an assessment of the immunogenicity and protective efficacy of *B. suis* OMVs in a murine model. Further studies are needed to determine the long-term immunity and effectiveness of OMVs as vaccines in the natural host.

## Data Availability

The original contributions presented in the study are included in the article/**Supplementary Material**, further inquiries can be directed to the corresponding authors.
