# Corrigendum: *Brucella suis* Δ*mapB* outer membrane vesicles as an acellular vaccine against systemic and mucosal *B. suis* infection

**DOI:** 10.3389/fimmu.2025.1565688

**Published:** 2025-02-05

**Authors:** Florencia Muñoz González, Magali G. Bialer, Maria L. Cerutti, Silvia M. Estein, Lila Y. Ramis, Pablo C. Baldi, Ángeles Zorreguieta, Mariana C. Ferrero

**Affiliations:** ^1^ Facultad de Farmacia y Bioquímica, Cátedra de Inmunología, Universidad de Buenos Aires, Buenos Aires, Argentina; ^2^ Instituto de Estudios de la Inmunidad Humoral (IDEHU), CONICET-Universidad de Buenos Aires, Buenos Aires, Argentina; ^3^ Fundación Instituto Leloir (FIL), IIBBA-CONICET (CONICET-FIL), Buenos Aires, Argentina; ^4^ Centro de Rediseño e Ingeniería de proteínas (CRIP), Universidad Nacional de San Martín, Buenos Aires, Argentina; ^5^ Laboratorio de Inmunología, Departamento de Sanidad Animal y Medicina Preventiva (SAMP), Centro de Investigación Veterinaria Tandil (CIVETAN-CONICET-CICPBA), Facultad de Ciencias Veterinarias (FCV), Universidad Nacional del Centro de la Provincia de Buenos Aires (UNCPBA), Tandil, Buenos Aires, Argentina; ^6^ Departamento de Química Biológica, Facultad de Ciencias Exactas y Naturales, Universidad de Buenos Aires, Buenos Aires, Argentina

**Keywords:** outer membrane vesicles, *Brucella suis*, vaccine, TAM, respiratory infection

In the published article, an author name was incorrectly written as “Maria L. Cerruti”. The correct spelling is “Maria L. Cerutti”.

The authors apologize for this error and state that this does not change the scientific conclusions of the article in any way. The original article has been updated.

