# Supplementary material for: Brucella suis ΔmapB outer membrane vesicles as an acellular vaccine against systemic and mucosal B. suis infection
Source: Front Immunol. 2025 Jan 20;15:1501791. doi: 10.3389/fimmu.2024.1501791 (PMC11788153; doi:10.3389/fimmu.2024.1501791)
Supplement: Supplementary file 1 [file DataSheet1.pdf]

Suplementary Table 1: OMVs associated proteins

Protein ID	Gene code	Gene name	Description
Q45689	BR0701	omp25	25 kDa outer-membrane immunogenic protein (omp25)
A0A0H3G433	BR1622	omp31b	Outer membrane protein Omp31 (omp31)
A0A0H3GC63	BR1284	omp22	OMP_b-brl domain-containing protein, outer membrane immunogenic protein
A0A0H3G4S3	BR0119	omp25c	Outer membrane immunogenic protein
A0A0H3G988	BR2149	dps	DNA starvation/stationary phase protection protein Dps
A0A0H3G827	BR1336		Acid-shock protein, putative
P0A3U5	BRA0423	omp31	31 kDa outer-membrane immunogenic protein
A0A0H3G3C6	BR1163		CaMKII_AD domain-containing protein
A0A0H3G6C2	BR0827		Uncharacterized protein
A0A0H3G313	BR0971		Outer membrane protein, putative
P64025	BR1251	tufA	Elongation factor Tu
Q8G1M4	BR0689	pepA	Probable cytosol aminopeptidase
A0A0H3G3Y9	BR1562		Membrane protein, putative
Q8G079	BR1230	rplB	50S ribosomal protein L2
A0A0H3G1U5	BR0335		Uncharacterized protein
P66434	BR1824		30S ribosomal protein S16
Q8G0G3	BR1132	eno	Enolase
Q8FZ07	BR1697	tolB	Tol-Pal system protein TolB
P66502	BR2185	rpsT	30S ribosomal protein S20
A0A0H3G398	BR0888		M24/M37 family peptidase
A0A0H3GDW5	BR2160	metK	Methionine adenosyltransferase
Q8G090	BR1214	rplO	50S ribosomal protein L15
Q8G082	BR1224	rpsQ	30S ribosomal protein S17
Q8G376	BR0074	argG	Argininosuccinate synthase
P66102	BR2120	rplT	50S ribosomal protein L20
Q8G0I5	BR1108	clpX	ATP-dependent Clp protease ATP-binding subunit ClpX
Q8FW95	BRA0565	bfr	Bacterioferritin

Q8FYA4	BR1981	argH	Argininosuccinate lyase
A0A0H3G1B0	BR0054		DUF1775 domain-containing protein
Q8FX87	BRA0195	groEL	Chaperonin GroEL
A0A0H3G7F5	BRA0909	purU	Formyltetrahydrofolate deformylase
P0A3P2	BR1930	omp19	Outer membrane lipoprotein omp19
A0A0H3G794	BR1164		conserved hypothetical protein
Q8G353	BR0099	ilvD	Dihydroxy-acid dehydratase
Q8G088	BR1217	rplR	50S ribosomal protein
A0A0H3G7V2	BRA0858	rbsB	Ribose ABC transporter, periplasmic D-ribose-binding protein
A0A0H3G3E6	BR1204		OmpA family protein
Q8FY12	BR2079	hslU	ATP-dependent protease ATPase subunit HslU
Q8G1C9	BR0790	rpsI	30S ribosomal protein S9
A0A0H3G5C0	BR1828		Lytic murein transglycosylase, putative
P0A3S8	BR1695	omp16	Peptidoglycan-associated lipoprotein
A0A0H3G939	BR2064	rho	Transcription termination factor Rho
A0A0H3G5J9	BR0496		Twin-arginine translocation pathway signal sequence domain-containing protein
A0A0H3G1F5	BR0118	omp25d	Outer membrane protein, putative
A0A0H3G4I7	BR1469		conserved hypothetical protein
A0A0H3G2L4	BR0708		conserved hypothetical protein
A0A0H3G678	BRA0088	modA	Molybdenum ABC transporter, periplasmic molybdenum-binding protein
Q8G077	BR1232	rplD	50S ribosomal protein L4
A0A0H3GAR7	BR0633		conserved hypothetical protein
Q8G3D4	BR0014		Leu/Ile/Val-binding protein homolog 3
A0A0H3GD35	BR1755		17 kDa surface antigen , putative lipoprotein
P59180	BR1227	rpsC	30S ribosomal protein
Q8G066	BR1247	rplA	50S ribosomal protein L1
A0A0H3G832	BR1579		Glycine betaine/L-proline ABC transporter, periplasmic glycine betaine-binding protein, putative
A0A0H3GA18	BRA0282		Pseudoazurin
A0A0H3G6N4	BRA0632		Amino acid ABC transporter, periplasmic amino acid-binding protein

A0A0H3GBL0	BR1011		DUF2059 domain-containing protein
A0A0H3G672	BR0486		Putative lipoprotein
Q8FXT0	BR2168	rpsO	30S ribosomal protein S15
A0A0H3G844	BR1596		conserved hypothetical protein
Q8FYR5	BR1799	atpD	ATP synthase subunit beta
A0A0H3GAY3	BRA0682	fliY	Amino acid ABC transporter, periplasmic amino acid-binding protein
P66827	BRA0703	sodC	Superoxide dismutase [Cu-Zn]
A0A0H3GA04	BRA0265		Sugar ABC transporter, periplasmic sugar-binding protein, putative
P64049	BR1161	tsf	Elongation factor Ts
A0A0H3G484	BR1693	cpoB	Cell division coordinator CpoB
A0A0H3G980	BR2138		Phosphate ABC transporter, phosphate-binding protein
A0A0H3G685	BR0772		SmpA_OmlA domain-containing protein
A0A0H3G576	BRA0011		Uncharacterized protein
P61713	BRA0695	ribH2	6,7-dimethyl-8-ribityllumazine synthase 2
Q8FVC3	BRA0921		Outer surface protein
A0A0H3G900	BR1761		Peptidase, putative
A0A0H3G2X2	BR0737		Putative lipoprotein
Q8FYF4	BR1927	mdh	Malate dehydrogenase
A0A0H3G608	BR0684		Peptidyl-prolyl cis-trans isomerase, putative
A0A0H3G5K6	BR0213		ABC transporter, periplasmic substrate-binding protein, putative
Q8FWR4	BRA0381		Periplasmic iron binding protein
Q8FW10	BRA0655	ugpB	sn-glycerol-3-phosphate-binding periplasmic protein UgpB
A0A0H3G9A1	BR2175		YaeC family lipoprotein
A0A0H3G5T3	BR2053		ABC transporter, periplasmic substrate-binding protein, putative
A0A0H3GFL4	BRA0738		Glycine betaine/L-proline ABC transporter, periplasmic glycine betaine-binding protein, putative
A0A0H3GAL2	BRA0538		Oligopeptide ABC transporter, periplasmic oligopeptide-binding protein
Q8G0J9	BR1094	ppi	Probable peptidyl-prolyl cis-trans isomerase
A0A0H3GC28	BRA0960		Lipoprotein , YaeC family
A0A0H3G6N1	BR0955		Amino acid ABC transporter, periplasmic amino acid-binding protein

A0A0H3G505	BR1689		Outer surface protein
Q8FUM7	BRA1193		Leu/Ile/Val-binding protein homolog 5
A0A0H3G8P9	BR1837		Carboxyl-terminal protease
A0A0H3G6A1	BRA0115		ABC transporter, periplasmic substrate-binding protein
A0A0H3G6H2	BRA0537		Oligopeptide ABC transporter, periplasmic oligopeptide-binding protein
P0A3N9	BRA0077	omp10	Outer membrane lipoprotein omp10
A0A0H3G425	BR1612		Putrescine-binding periplasmic protein
A0A0H3G8C6	BR1468		Choloylglycine hydrolase family protein
Q8FW84	BRA0576		peptide ABC transporter, periplasmic peptide-binding protein
A0A0H3GFI5	BRA0700		Iron compound ABC transporter, periplasmic iron compound-binding protein, putative
A0A0H3G9E5	BR0048		Translocation and assembly module TamA
A0A0H3GBL9	BR1022		Putative lipoprotein
A0A0H3GFM9	BRA0756		Iron compound ABC transporter, periplasmic iron compound-binding protein
A0A0H3G7C2	BR1205		Putative lipoprotein
A0A0H3G234	BR0440		MliC domain-containing protein
A0A0H3G9B9	BR0010		ABC transporter, periplasmic substrate-binding protein, putative
A0A0H3G440	BR1194		TRAP transporter solute receptor TAXI family protein
A0A0H3G825	BR1330		Sulfate ABC transporter, sulfate-binding protein, putative
A0A0H3GBL5	BRA0937		Sugar ABC transporter, periplasmic sugar-binding protein

Table S1: OMVs associated proteins detected by nano LC-MS/MS coupled to a QExactive Mass Spectrometer produced by *B. suis* 1330 (wt) and Δ*mapB* strains.
